# Federated Subgraph Learning via Global-Knowledge-Guided Node Generation

**DOI:** 10.3390/s25072240

**Published:** 2025-04-02

**Authors:** Yuxuan Liu, Zhiming He, Shuang Wang, Yangyang Wang, Peichao Wang, Zhangshen Huang, Qi Sun

**Affiliations:** 1 School of Information and Communication Engineering, University of Electronic Science and Technology of China, Chengdu 611731, China; 202311012213@std.uestc.edu.cn (Y.L.); peichaowang@std.uestc.edu.cn (P.W.); zsh@alu.uestc.edu.cn (Z.H.); 2 Hangzhou Nuowei Information Technology Company Ltd., Hangzhou 310059, China; qi.sun2166@nvxclouds.com; 3 School of Aeronautics and Astronautics, University of Electronic Science and Technology of China, Chengdu 611731, China; yangyangw@uestc.edu.cn; 4 Shanxi Province Key Laboratory of Intelligent Detection Technology & Equipment, North University of China, Taiyuan 030051, China

**Keywords:** federated learning, distributed learning, graph learning, machine learning, deep learning

## Abstract

Federated graph learning (FGL) is a combination of graph representation learning and federated learning that utilizes graph neural networks (GNNs) to process complex graph-structured data while addressing data silo issues. However, during the local training of GNNs, each client only has access to a subgraph, significantly deteriorating performance. To address this issue, recent solutions propose completing the subgraph with pseudo graph nodes generated by a generator trained using the local subgraph. Despite their effectiveness, such methods may introduce biases as the local pseudo graph nodes cannot accurately represent the global graph distribution. To overcome this problem, we introduce MN-FGAGN, which mitigates the impact of missing neighbor information by generating pseudo graph nodes that follow the global distribution. The main idea of our approach is to partition the generative adversarial neural network into a client-side discriminator and a server-side generator. In this way, the generator can receive supervised information from all clients and can thus generate graph nodes that contain global information. Experiments on four real-world graph datasets show that it outperforms the state-of-the-art methods.

## 1. Introduction

Federated graph learning (FGL) is a novel type of distributed graph neural network (GNN) with privacy-preserving capabilities. In contrast to distributed GNNs, it can collaboratively train a global neural network without revealing the local data of clients (e.g., mobile phones, computers, and other edge devices [1,2,3,4]), enabling different clients to benefit from it. It is widely applied in various fields such as social networks [5,6], health records [7,8], blockchain [9], and anomaly detection [10]. In this paper, we focus on the node classification problem in FGL.

**Figure 1:**
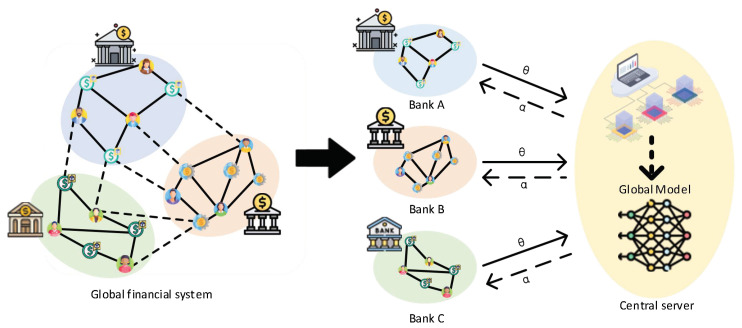
For example, in real life, the purchase relationship between a person and a financial product from a bank constitutes a bipartite graph. The global graph records the users (class A nodes of the bipartite graph) and financial products (class B nodes of the bipartite graph) and the demand relationships (edges) between them at a certain time. Specifically, it represents the distributed storage of the global graph of the financial system in such a way that the subgraph division leads to the absence of the originally existing edges (virtual edges) due to privacy protection requirements.

In traditional approaches, clients on different edges possess independently complete graph data [11,12]. They enhance the classification accuracy of the global model through client-side weighting or data augmentation. However, when dealing with large-scale graph data, the data load capacity of clients often cannot accommodate an entire graph (global graph), resulting in local data from different edge devices being a subset of the global graph. As clients do not communicate with each other, this inevitably leads to data loss issues in the connections of the subgraphs among different clients (as shown in Figure 1), further impacting the classification accuracy of local GNNs and the performance of the global model. To address these issues, FedSage+ [13] and FedNI [14] employ generative network collaboration to recover lost subgraph data; the LLCG [15] model uses local learning and global correction methods to enhance model performance; PEARL [7] integrates self-supervised learning into the federated graph learning framework to enhance the global model’s performance. However, they all overlook the local bias present in local subgraphs compared to the global graph [16], which leads to recovered subgraph data being more inclined towards local data (local biases).

In contrast to them, in this paper, we focus on enhancing the quality of subgraph data at the client side, eliminating local biases in the generated subgraph data, and thereby improving the performance and classification accuracy of the global aggregation model. Specifically, we aim for the missing data in the subgraphs generated on the client side to incorporate global knowledge, which requires implicitly injecting global knowledge into different clients. However, due to the different statistically heterogeneous distributions of graph data across different clients, the aggregated global model may even perform worse than individual client data [17]. Simply injecting the aggregated global knowledge into the clients often introduces additional biases. To address this challenge, inspired by game theory, we utilize a generator in the server and discriminators on each client to jointly generate global nodes by computing the similarity between local node data and global node data, selecting the most similar global knowledge to fuse with local knowledge, and achieving personalized injection of global knowledge to eliminate biases in private data.

Based on the above motivation, we propose a novel GNN method called MN-FGAGN (**M**issing **N**eighbor Generation with **F**ederated **G**enerative **A**dversarial **G**raph **N**etworks). This method aims to generate unbiased missing subgraph data, thereby enhancing the quality of local data and consequently improving the model. Specifically, we have devised a global generation strategy utilizing a global generator to produce data closely resembling the locally missing nodes. It employs a central server generator to learn the feature distribution of each device and generate nodes, with local discriminators on the devices for node selection and mending, thereby reducing the risk of information leakage. Furthermore, it employs federated generative adversarial graph networks to learn the feature distribution of each local subgraph, generating nodes with global knowledge to better mimic the characteristics of real missing nodes, ultimately eliminating local biases. We conducted extensive experiments on various datasets, demonstrating that FGAGN outperforms various baselines. Our method presents the following specific advantages:

- We propose a novel method called MN-FGAGN, which employs an innovative global generation strategy. This strategy personalizes the injection of global knowledge to eliminate the local biases present in generated data. By enhancing the local node representations and the quality of local data, our approach improves the node classification capability of FGL.
- Our method utilizes the value of the loss function to transmit global knowledge. Compared to transferring data and model parameters, our approach offers enhanced privacy.
- We conducted experiments on four real-world datasets, and our method achieved superior results compared to existing approaches.

## 2. Related Works

### 2.1. Federated Learning

Federated learning has emerged as a privacy-preserving paradigm that facilitates the collaborative training of a global model using distributed training data across multiple devices [18,19,20,21,22]. In distributed machine learning and federated learning, most approaches to improve communication efficiency involve reducing communication time at the expense of computational effort [23,24,25]. However, these approaches are primarily based on independent and identically distributed (IID) data for experiments. In federated learning, data are often non-IID and distributed, which can result in significant performance degradation [26,27]. To address the issue of non-IID data, personalized federated learning [28,29] and meta-learning [30,31] have been proposed. For enhancing data protection, various algorithms, such as differential privacy (DP) [32,33] and secure multi-party computation (MPC) [34], are employed.

### 2.2. Federated Graph Learning

FGL is an intersection of federated learning and graph representation learning. FedGraphNN [11] introduces a novel combined framework of federated learning and GNNs. Clustering in FedCG [35] addresses statistical heterogeneity, enabling graph convolutional networks to share knowledge. The GCFL+ algorithm [36] dynamically identifies clusters of local systems through the utilization of the gradient of the GNN, and it has been theoretically proven that these clusters can effectively mitigate structural and feature heterogeneity among the graphs belonging to the local systems. Recently, the Alibaba Dharma Institute has integrated research pertaining to GNNs in various federated scenarios and has established a research platform known as FederatedScope [37] to further these efforts. In contrast to existing FGL paradigms, FedSage+ [13] presents a new contextual problem: how to enhance the performance of individual device models in FGL when devices have missing information in their subgraph sections. FedSage+ trains a missing neighbor generator alongside FedSage to handle missing links across local subgraphs. FedNI [14] optimizes this approach by proposing the use of generative adversarial networks (GANs) on each device, generating local network information that closely resembles the distribution of subgraph data. LLCG [15] utilizes distributed learning, with each local model relying on a global graph stored by a trusted third-party server for modification. This approach considers generating local subgraphs that closely resemble real situations in the absence of subgraph information but struggles to generate high-quality subgraphs with global knowledge while protecting node data privacy.

## 3. Problem Formulation and Preliminaries

### 3.1. Graph Neural Network

The GNN is a neural network model for graph structure data learning, which represents the nodes and edges in a graph as vectors or matrices and learns their representations through neural network models, e.g., recursive GNN, convolutional GNN, graph self-encoder, etc. [38], thus enabling the learning of the whole graph. Specifically, GNNs can be viewed as a method for iteratively updating node representations. For an undirected graph G=(V,E) with *n* nodes and *m* edges, suppose we have initialized a *d*-dimensional vector representation $h_{v}^{\left(0\right)}\in R^{d}$ for each node v∈V, where $h_{v}^{\left(0\right)}$ denotes the vector representation of node *v* at the 0—th level. The main goal of the GNN is to obtain a vector representation $h_{v}^{\left(L\right)}$ that captures the semantic information of the entire graph by iteratively updating the vector representation of each node, where *L* denotes the total number of layers of the GNN.

Each layer of the GNN can be represented as the following update rule:

(1) $$h_{v}^{\left(l+1\right)}=\sigma \left(\sum_{u\in N(v)}\frac{1}{c_{v,u}}W^{\left(l\right)}h_{u}^{\left(l\right)}+b^{\left(l\right)}\right)$$

where $h_{u}^{\left(l\right)}$ denotes the hidden representation of node u at the lth level of the GNN, N(v) denotes the set of neighboring nodes of node v, $W^{\left(l\right)}$ and $b^{\left(l\right)}$ denote the weight matrix and bias vector at the *l*th level, respectively, σ(·) is an activation function, and $c_{v,u}$ is a normalization factor that can be used to normalize the node degrees, with the effect of making the node degrees on the representation more balanced. This equation can be viewed as a weighted sum of the vector representations of the neighboring nodes with a bias vector, which is then nonlinearly transformed by the activation function σ(·) to obtain the new vector representation. By continuously and iteratively updating the vector representation of each node, the final vector representation $h_{v}^{\left(L\right)}$ obtained by the GNN can be used for various graph structure data learning tasks, such as node classification, graph classification, link prediction [39], etc.

### 3.2. Federated Learning of Graph Neural Network

The main scope of the federated GNN does not share data to co-train a global GNN model. For the problem of missing subgraph information, existing methods train a local generator jointly to extend local subgraph data based on local subgraph information and subgraph information on other clients. The objective function of the joint generator [13] is

(2) $$\begin{array}{cc} L_{i}^{f} & =\frac{1}{|{\bar{V}}_{i}|}\sum_{v\in {\bar{V}}_{i}}\sum_{p\in [{\tilde{n}}_{v}]}min_{u\in N_{G_{i}}\left(v\right)\cap V_{i}^{h}}\left(|\right|{\tilde{x}}_{v}^{p}-x_{u}{\left|\right|}_{2}^{2})\\ \; & +\alpha \sum_{j\in [M]/i}min_{u\in V_{j}}\left(|\right|H_{i}^{g}{\left(z_{v}\right)}^{p}-x_{u}{\left|\right|}_{2}^{2}) \end{array}$$

where $x_{v}^{p}\in R$ denotes the predicted Pth node feature and $u\in V_{j}$ denotes the nearest node to $G_{i}$, thus to model the missing node information in the local subgraph $G_{i}$. This enhancement method requires sharing data node information across clients, which may violate the privacy constraint in the federated learning setting that users cannot interact [40,41]. Also, the information encoded in the local neighbor generator is limited to and biased towards this map, which prevents it from truly generating neighbors belonging to other data owners and connected by missing cross subgraphs.

### 3.3. Generative Adversarial Network

Generative adversarial networks (GANs) [42] are generative models based on the idea of game theory, where two neural network models, i.e., a generator and discriminator, are trained to play against each other iteratively to generate samples similar to the distribution of real data. The goal of the generator is to generate fake samples that are similar to the distribution of real data so that the discriminator cannot accurately distinguish between real and fake samples, while the goal of the discriminator is to accurately distinguish between real data and fake samples generated by the generator, thus helping the generator to learn to generate more realistic samples. The generator and the discriminator iterate through adversarial learning, and eventually the generated samples become more and more realistic. The core idea of generating an adversarial neural network (GAN) is

(3) $$\begin{array}{cc} \min_{G} & \max_{D}L\left(D,G\right)=E_{x∼P_{data}\left(x\right)}\left[logD\left(x\right)\right]\\ \; & +E_{z∼p_{z}\left(z\right)}\left[log\left(1-D\left(G\left(z\right)\right)\right)\right] \end{array}$$

where D(x) denotes the discriminator’s prediction of sample *x*, G(z) denotes the fake sample obtained by the generator by mapping random noise *z* into the data space, $p_{data}\left(x\right)$ denotes the true data distribution, and $p_{z}\left(z\right)$ denotes the noise distribution. By training the generator and discriminator alternately, GANs can continuously generate more realistic fake samples to simulate the real data distribution, which makes GANs widely used in generating images, audio, etc.

### 3.4. Problem Setup

Table 1 summarizes the key symbols used throughout this paper for easier reference and improved readability.

In the context of federated learning, multiple clients possess local subgraphs that collectively form a global graph. We provide a formal definition for this global graph as follows: Consider a global undirected graph G={V,E,F}, where *V* represents the set of nodes, *E* represents the set of edges, and *F* represents the nodes feature. Suppose there are $N_{c}$ disjoint subsets $G_{n}=\left\{V_{n},E_{n},F_{n}\right\}\left(n\in N_{c}\right)$ and $E_{G_{i},G_{j}}\left(i,j\in N_{c}\right)$ represents the set of missing edges between subgraphs $G_{i}$ and $G_{j}$. They have the following relationships:

- ${⋃}_{i=1}^{N_{c}}V_{i}=V$: the union of the node sets of each subgraph is the node set of the global graph.
- ${⋃}_{n}^{N_{c}}E_{n}+{⋃}_{i,j}^{N_{c}}E_{G_{i},G_{j}}=E$: the union of the edge sets of each subgraph and the union of the sets of missing edges between subgraphs form the set of edges of the global graph.
- $\forall i,j\in 1,2,\cdots ,N_{c}$, if i≠j, then $V_{i}\cap V_{j}=\emptyset$: each vertex belongs to only one subset.
- If *a* and *b* are nodes at the ends of the missing edge $e_{a,b}$, where edge $e_{a,b}\in E_{G_{i},G_{j}}$, node $a\in V_{i}$, and node $b\in V_{j}$, then nodes *a* and *b* are missing nodes for the subgraphs $G_{i}$ and $G_{j}$, respectively.

Under these conditions, the union of the $N_{c}$ subgraphs and the set of missing edges constitutes the global graph, which can be represented as $G={⋃}_{n}^{N_{c}}G_{n}+{⋃}_{i,j}^{N_{c}}E_{G_{i},G_{j}}$.

It is worth noting that the term “subgraph” in this context refers to a subset of vertices and edges, rather than the traditional notion of a subgraph of the global graph. Thus, the vertices in $G_{n}$ must be a subset of *V*, and the edges in $G_{n}$ must be a subset of the edges in *E* that connect the vertices in *V*.

**Goal**: We formally express this problem as the task of discovering a method to learn the minimization of the empirical loss *W* for as many client distribution generators as possible. This can be mathematically represented as

(4) $$\begin{array}{cc} W(G)= & \frac{1}{N_{c}}\sum_{n=1}^{N_{c}}E_{\left(G_{n},Y_{n}\right)∼S_{n}}\left[L_{n}\left(E_{z∼GP}\left(G\left(z\right)\right),y_{n}\right)\right]\\ \; & where\;\hat{G}=\arg\min W\left(G\right) \end{array}$$

where *G* represents the global knowledge data generator and W(P) is the global empirical loss that we try to minimize. $L_{n}$ denotes the local loss function of client *n* and $E_{\left(G_{n},Y_{n}\right)∼S_{n}}$ denotes the expectation on the data distribution $S_{n}$ of client *n* and $\left(G_{n},Y_{n}\right)$ is the graph $G_{n}$ and its corresponding label $Y_{n}$ drawn from the data distribution of client *n*. $E_{z∼GP}$ means that noise z obeys a Gaussian distribution and $y_{n}$ is the ground truth.

## 4. System Model

In this section, we propose a novel machine learning framework, MN-FGAGN, that addresses the dual challenges of preserving privacy and generating nodes with global knowledge in the context of missing subgraph neighborhood completion.

### 4.1. Overview

**Figure 2:**
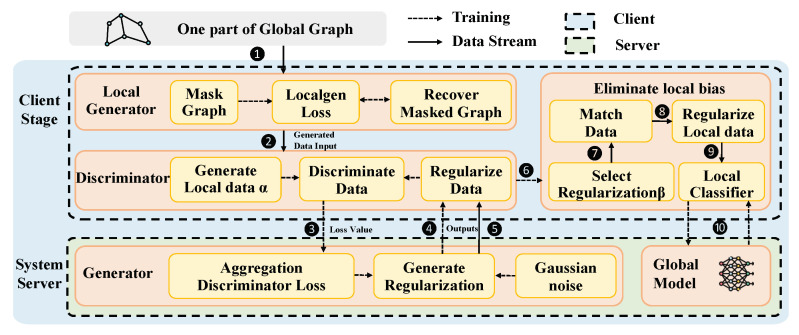
Overview of MN-FGAGN system architecture and modules.

The MN-FGAGN framework comprises a central server (*S*) and $N_{c}$ clients, forming a federated learning network, with the server consisting of a generation module ($G_{global}$) and an aggregation module (Agg(·)), while the client consists of a local node generator (Localgen), a discriminator ($D_{n}$), and a node classifier (Classifier) (Figure 2). In this subsection, we present an overview of the entire process.

Initially, the original subgraph is masked and fed to the Localgen (❶), which trains the Localgen by recovering the masked subgraph. Subsequently, the trained Localgen generates the missing neighbors (❷), resulting in the set of generated nodes denoted as α. However, this approach may introduce local bias in the node data as Localgen can only generate missing data based on local subgraphs.

During the training phase, the generator uses Gaussian noise as input to produce global knowledge data (❹), and the discriminator ($D_{n}$) uses the locally generated node embeddings as the “ground truth” to evaluate the global knowledge generated by the generator. The discriminator’s loss function values $L_{n}$ are then transmitted to the central server (❸). At the server, the discriminator’s losses are aggregated across all clients to guide the generator in generating the global knowledge. The generator then transmits the generated global knowledge to the clients to guide discriminator training (❹). This process continues until a balance is achieved between the generator loss and the aggregated discriminator loss. The global knowledge is a global regularization term generated after learning the distribution of as much client data as possible, which helps local nodes refine the representation without compromising privacy.

Once the generator’s training is complete, the generator sends the global knowledge to all users (❺). The user then employs a discriminator to select the $N_{g}^{n}$ data with the highest discriminant probability among them (❻), which are used as the β set ($N_{g}^{n}$ denotes the number of missing nodes generated by the Localgen module of client *n*). For any node $\beta_{i}$ in the set of β nodes with the set of nodes $\alpha_{j}$ generated by Localgen, the feature distance *d* is computed. The pair $\left(\beta_{i},\alpha_{j}\right)$ that minimizes $d(\beta_{i},\alpha_{j})$ is considered as the matched data (❼). The matched data $\beta_{i}$ are then used as global knowledge for feature fusion of the original node $\alpha_{i}$, eliminating the inherent bias of the local dataset and enhancing the feature representation of the generated node (❽). Finally, the corrected subgraph is used as input to the classifier (❾), and the parameters of the local classifier are updated using the FedAvg algorithm (❿).

### 4.2. MN-FGAGN

In the context of federated learning, the issue of missing information in cross-domain subgraphs poses a significant challenge. Conventional algorithms attempt to generate missing nodes based on local subgraphs, potentially resulting in node bias problems. To mitigate this issue, we introduce a novel framework for FGL. Our approach aims to eliminate local node bias by injecting global knowledge with a federated generative neural network. This network guides the server-side generator to produce data that effectively regularize local nodes, thereby enhancing the accuracy and adequacy of local node representation.

**Figure 3:**
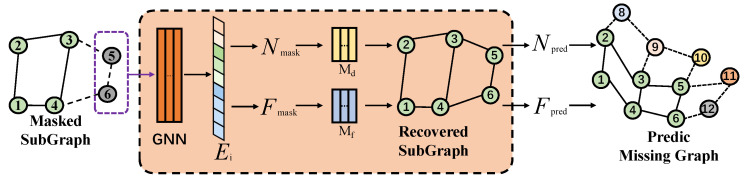
The training process for Localgen involves three frameworks: a GNN module and two MLP modules. The GNN module extracts feature information from the graph, while $M_{d}$ predicts the number of missing nodes and $M_{f}$ predicts their features.

**Module description of Localgen:** In traditional GNNs, it is common to construct a local subgraph centered around a specific node to analyze the relationships between that node and its directly connected nodes. This type of local subgraph constructed from a central node is referred to as an ego-graph. The goal of Localgen is to generate missing neighboring nodes of the ego-graph in the local context, as illustrated in Figure 3. To achieve this, we adopt a methodology similar to that proposed in Zhang et al. [13], which generates missing nodes to serve as local learning objects for subsequent generators. Localgen comprises three components: (i) a GNN that extracts features of *i* nodes and their neighbors within the *k*-hop range of the ego-graph; (ii) a multilayer perceptron ($M_{d}$) that predicts the extent of missing nodes; and (iii) another multilayer perceptron ($M_{f}$) that predicts the features of missing nodes. In particular, we first mask the original subgraph $G_{n}$ of the *n*th client and input the ego-graph centered on node *i* into the GNN for feature extraction. The output of the GNN is the feature matrix $E_{i}$ of the ego-graph. Subsequently, we send $E_{i}$ into $M_{d}$ and $M_{f}$. $M_{d}$ uses $N_{mask}$ as the ground truth to predict the number of neighbors masked by node *i*, while $M_{f}$ uses $V_{mask}$ as the ground truth to predict the features of the nodes masked by node *i*’s neighbors. After training Localgen, we use it to generate missing nodes $V_{pred}$. Specifically, we obtain edges for the *i*th node from the missing nodes predicted by $M_{d}$ and use $M_{f}$ to generate their features. We then employ these generated nodes and features to repair the original subgraph. The loss function of Localgen is as follows:

(5) $$\begin{array}{cc} L_{gen} & =l\left(\phi |N_{mask},N_{pred}\right)+l\left(\phi |V_{mask},V_{pred}\right)\\ \; & =-[N_{pred}\log N_{mask}+\left(1-N_{pred}\right)\log\left(1-N_{mask}\right)]\\ \; & -[V_{pred}\log V_{mask}+\left(1-V_{pred}\right)\log\left(1-V_{mask}\right)] \end{array}$$

However, the approach of generating missing information locally has some limitations. One of the main issues is that the missing nodes generated by the local generator are primarily based on the missing data predicted by the local subgraph. As a result, the data distribution is skewed towards the nodes of the local subgraph and deviates from the actual node distribution. This can lead to biased generated data. To address this limitation, we propose a method to inject global knowledge into the generated local subgraphs to eliminate local biases, which will be described in the following two subsections.

**Global knowledge generator ($G_{global}$):** To address the issue of biased local missing nodes generated by the local generator, we employed the MN-FGAGN method. This method involves utilizing $G_{global}$ to generate global knowledge, thereby improving the adequacy of the subgraph representation and proximity to actual missing data. Specifically, for $G_{global}$, we first used noise *Z* to generate $N_{g}^{all}=\left\{N_{g}^{all}\right|N_{g}=\frac{1}{2}\sum_{n=1}^{N_{c}}\sum_{i=1}^{|V_{n}|}degree\left(v_{i}\right)$, $v_{i}\in V_{n},n\in N_{c}\}$ data of dimension *T* through a fully connected neural network. Here, *T* denotes the node’s feature dimension, which is identical to the feature dimension $F_{i}$ generated by Localgen. Moreover, $N_{g}^{all}$ indicates the total number of missing nodes on all clients, degree(·) refers to the number of missing node degrees, and $|V_{n}|$ represents the total number of *n* clients. Next, the input layer receives the noise in *T* dimensions, and the output layer generates the feature matrix $O_{n}\in R^{N_{g}\times T}$ in dimensions. The final softmax function is applied to normalize each output vector as

(6) $$Y_{gn}=softmax\left(O_{n}\right)=\frac{E(O_{n})}{\sum_{n=1}^{N_{c}}E\left(O_{n}\right)},\;\mathrm{for}\;n=1,\cdots ,N_{g}$$

where the rows of matrix $O_{n}$ represent the number of generated regularization terms, and the column vectors of matrix $O_{n}$ represent the features corresponding to the missing nodes.

**Distribution discriminator ($D_{n}$):** In the MN-FGAGN framework, the discriminator is actually a distributed discriminator that evaluates whether the global knowledge generated by the generator is similar to the local node data distribution; each client discriminator $D_{n}$ computes a local loss function value and sends it to the central server for global aggregation to guide the generator to generate more realistic data.

**Figure 4:**
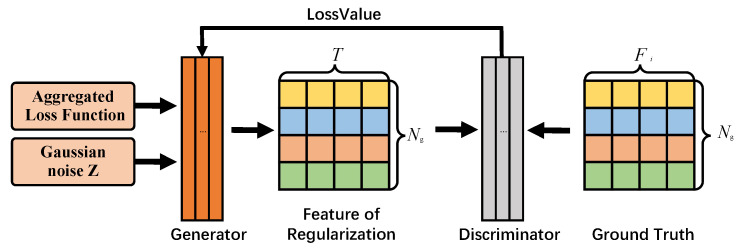
The training process for the generator and discriminator involves setting up a separate discriminator on each client, while the generator itself is hosted on the server. The discriminators assess the authenticity of the generated data and provide feedback to guide the generator’s training. This approach avoids the need for the original data to be transmitted to the server, thereby reducing the risk of privacy breaches.

**Joint training process:** In this section, we describe the joint training process of the generator $G_{global}$ and discriminator $D_{n}$ in MN-FGAGN. The generator’s core purpose is to learn the feature distribution of data sources on multiple clients to generate regulated data with global knowledge, which can correct the biased data generated by Localgen. The specific joint training process is as follows:

Initially, we build the global generator $G_{global}$ on the server and the discriminator $D_{n}$ on each device. As shown in Figure 4, $G_{global}$ generates global data by Gaussian noise. The central generator broadcasts a matrix $O_{n}$ containing information such as the characteristics of the generated nodes to each device. On the device, the discriminator generates the feature matrix $L_{n}\in R^{N_{g}\times F_{g}}$ of the missing data in Localgen, where the feature dimension $F_{g}$ of $L_{n}$ is the same as that of $O_{n}$. The purpose of the generator loss function is to minimize the prediction probability of the discriminator for the error samples and to make the generated error samples more realistic. The generator loss function can be defined as follows:

(7) $$\min_{G_{global}}L_{global}=E_{z∼p(z)}\left[\log\left(1-Agg\left(D_{n}\left(G_{global}\left(z\right)\right)\right)\right)\right]$$

where $E_{z∼p(z)}\left[log\left(1-Agg\left(D_{n}\left(G_{global}\left(z\right)\right)\right)\right)\right]$ represents the minimal probability that the false sample $G_{global}\left(z\right)$ generated by the generator is predicted by the discriminator as a true sample. In other words, the generator loss function aims to generate more realistic false samples to reduce the value of $\log(1-D_{n}\left(G_{global}\left(z\right)\right))$ and, in turn, minimize the generator loss function.

In the second stage, we stop updating the gradient of the discriminator $D_{n}$. The discriminator loss function aims to maximize its ability to discriminate between true and false samples, i.e., to maximize the probability of correctly predicting true samples while minimizing the probability of correctly predicting false samples. The discriminator loss function can be defined as follows:

(8) $$\begin{array}{cc} \max_{D_{n}}L_{D_{n}} & =E_{x∼p_{data}\left(x\right)}\left[\log Agg\left(D_{n}\left(x\right)\right)\right]\\ \; & +E_{z∼p(z)}\left[\log\left(1-Agg\left(D_{n}\left(G_{global}\left(z\right)\right)\right)\right)\right] \end{array}$$

where Agg(·) denotes the aggregation function output by the aggregation discriminator, and $E_{z∼p(z)}[\log\left(1-D_{n}\left(G_{global}\left(z\right)\right)\right]$ represents the highest probability that the spurious sample $G_{global}\left(z\right)$ generated by the generator is predicted as spurious by the discriminator. The discriminator loss function aims to generate stronger discriminators by maximizing the value of $\log(1-D_{n}\left(G_{global}\left(z\right)\right))$, thereby better identifying false samples. In this way, we can generate $N_{g}$ regularization terms, incorporating the personalized global knowledge of each missing node.

### 4.3. Regularization

**Figure 5:**
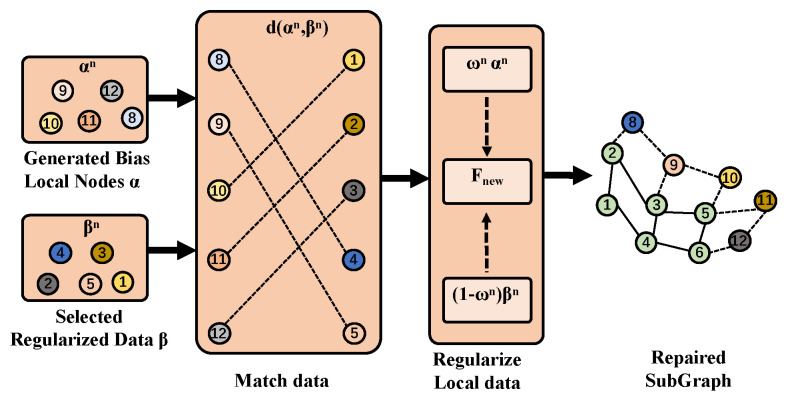
Two types of bias data are generated: α, which is based on the local subgraph, and β, which is regularized by the central generator for the client. The feature fusion process eliminates the feature bias in the local subgraph by matching the two types of data.

The regularized selected data are denoted by the regularization process in Figure 5. The specific steps are as follows:

**Selecting regularized data:** The missing node set generated by Localgen at the *n*th ($n\in N_{c}$) client is denoted by $\alpha^{n}$. The *n*th client selects the top $N_{g}^{n}$ generated data based on the output probability of discriminator $D_{n}$ as the candidate set $\beta^{n}$ of high-quality data, where $N_{g}^{n}$ is the degree of the missing node predicted by the Localgen of the *n*th client.

**Matching data**: The generated data feature representation $f_{\beta_{j}^{n}}$ and the real data feature representation $f_{\alpha_{i}^{n}}$ are extracted from the penultimate hidden layer of discriminator $D_{n}$. For each local node $\alpha_{i}^{n}$, the distance between its feature representation $f_{\alpha_{i}^{n}}$ and the feature representation $f_{\beta_{j}^{n}}$ of each generated data point in the high-quality generated data candidate set is calculated. The generated data point $\beta_{j}^{n}$ that minimizes the distance is found. That is, for each $\alpha_{i}^{n}$, $\beta_{j}^{n}$ is found, satisfying

(9) $$d(f_{\beta_{j}^{n}},f_{\alpha_{i}^{n}})=\arg\min||f_{\beta_{j}^{n}},f_{\alpha_{i}^{n}}{\left|\right|}_{2}^{2}\;\mathrm{where}\;i,j\in T_{\alpha^{n}},T_{\beta^{n}}$$

where *d* is a feature distance calculation function, and $T_{\alpha^{n}}$ and $T_{\beta^{n}}$ respectively represent the indices of the local nodes and the data indices of the selected high-quality data candidate set $\beta_{i}^{n}$. This approach ensures effective matching between local nodes and generated data while maintaining the quality of generated data.

**Regularizing data:** Feature fusion is performed between the missing node set $\alpha^{n}$ of the subgraph generated based on the local subgraph and the global knowledge $\beta^{n}$ from the central server. The specific formula is as follows:

(10) $$\begin{array}{cc} F_{new} & =\omega^{n}\alpha^{n}+\left(1-\omega^{n}\right)\beta^{n}\\ \; & =\left(\frac{D(\beta^{n})}{D\left(\beta^{n}\right)+D\left(\alpha^{n}\right)}\right)\times f_{\beta^{n}}+\left(\frac{D(\alpha^{n})}{D\left(\beta^{n}\right)+D\left(\alpha^{n}\right)}\right)\times f_{\alpha^{n}} \end{array}$$

where $F_{new}$ denotes the data after bias correction. This process eliminates the local deviation of the missing node set $\alpha^{n}$ in the subgraph, thereby improving the data quality of the local subgraph.

### 4.4. Classifier Training

Our approach consists of training a global node classification model using joint learning [40], and we feed the full subgraph of the repair to each client’s local classification model GraphSAGE [43].

For $N_{K}$ samples owned on *n* devices, the training objective is to minimize the loss function $L_{n}\left(\omega \right)$, where ω is the model parameter. Let $G_{n}$ be the dataset on the *n*th device, and $G=\{G_{1},G_{2},\cdots ,G_{N_{c}}\}$ be the set of data on all devices. Let $w_{t}$ be the server model parameters in the *t*th iteration and $w_{t,n}$ be the local model parameters on the *n*th device in the *t*th iteration. The workflow is (1) randomly select devices; (2) calculate the local loss function and gradient for each device; (3) compute the global gradient by averaging local gradients from selected devices and update model parameters; (4) repeat until the stopping condition is met.

This process allows the local classifiers to learn from each other and improve their performance on their respective subgraphs. The aggregated global models can then be used for node classification tasks on the entire graph.

In summary, our proposed framework consists of three main components: Localgen, which predicts locally missing nodes; $G_{global}$, which generates globally missing nodes; and a local classifier, which is trained on the repaired subgraphs using joint learning. This combination of components allows for an effective and efficient learning process that accommodates features of both local and global graphs, ultimately leading to an improvement in node classification performance.

### 4.5. Algorithms

In this study, we introduce a collaborative training workflow Algorithm 1), MN-FGAGN, to tackle challenges in distributed learning. The algorithm comprises four phases, which are structured as follows:

**Procedure Localgen:** Co-training of Localgen with masked and unmasked subgraphs. We adopt a local generator (Localgen) to efficiently extract data features through co-training with masked and unmasked subgraphs.

**Procedure DiscriminatorLossUpdate:** Learning distributed data features without the direct manipulation of original data. Our privacy-preserving approach enables the algorithm to learn the data’s feature distribution without accessing the original data, thus minimizing the risk of data leakage.

**Procedure ClientLocalUpdate:** The selection of matched regularization data and elimination of local missing data bias. We enhance the distributed data’s consistency and accuracy by selecting matched global knowledge and removing missing data bias, subsequently improving the global model’s performance.

Optimization of the global model through repaired subgraph co-training. The specific steps are divided into: *(1) Local computation*: randomly selecting devices from all available devices in the current iteration round (in this paper, for the sake of the repeatability of the experimental results, we chose full participation of equipment). For the chosen devices *n*, the local loss function $L_{n}\left(w_{t}\right)$ is computed using local data $G_{n}$, and the local gradient $\nabla L_{n}\left(\omega_{t}\right)$ is determined. *(2) Gradient aggregation*: the weighted averaging of the selected devices’ local gradients to obtain the global gradient ${\bar{g}}_{t}$. The model parameters are then updated as $\omega_{t+1}=\omega_{t}-\eta g_{t}^{*}$ using the global gradient ${\bar{g}}_{t}$, where η represents the learning rate, controlling the magnitude of model parameter updates.

By employing this collaborative training workflow, we effectively address distributed learning challenges and ensure normalized data generation, consequently enhancing the global model’s performance. Our approach offers an efficient solution for large-scale distributed learning problems while preserving data privacy. The MN-FGAGN algorithm is highly scalable and applicable to various distributed learning scenarios, including IoT, mobile devices, and data centers. Moreover, it can be integrated with other optimization strategies and regularization techniques to further improve the global model’s performance and stability.
**Algorithm 1:** MN-FGAGN algorithm
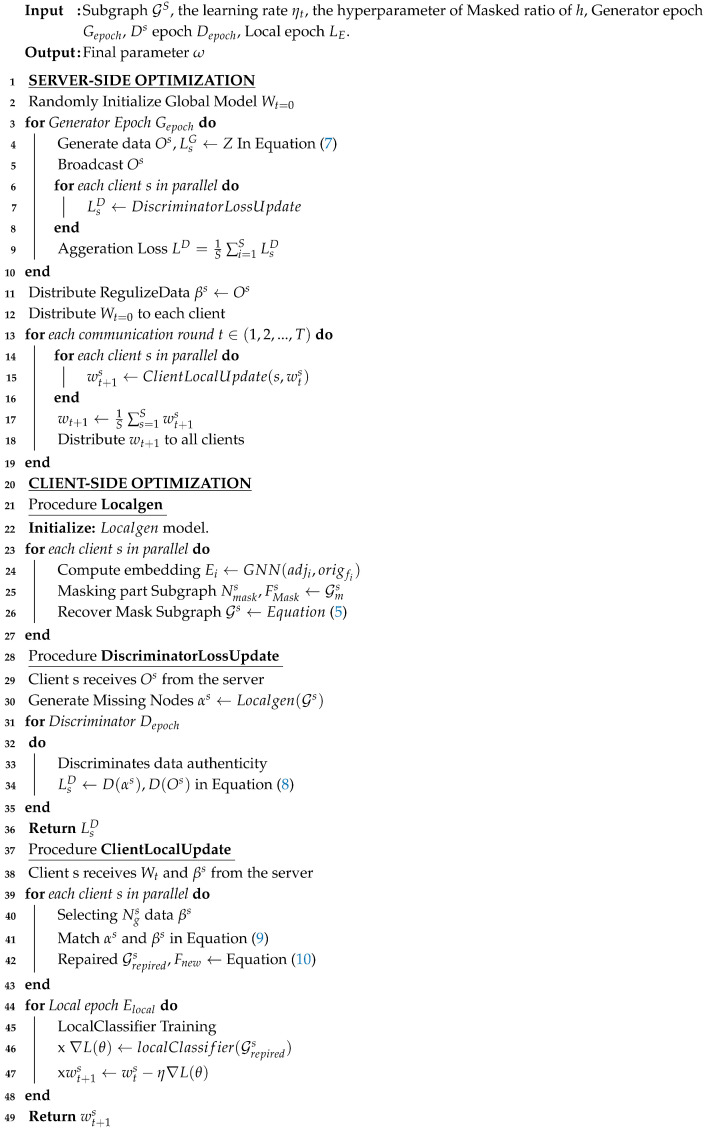


### 4.6. Discussion of MN-FGAGN

#### 4.6.1. Privacy Discussion

Compared to traditional FGL algorithms [13,36], MN-FGAGN transmits only the local discriminator’s loss value during the information transmission phase to guide the training of the central generator. First, the loss value from the local discriminator does not contain information about the local raw data, which inherently prevents privacy leakage. Second, during communication, both local data and the local model remain on the client side, which prevents attackers from reconstructing local data from the model. Finally, MN-FGAGN can be integrated with various privacy-preserving techniques, such as DP [32] and MPC [34], to further enhance the privacy of the FGL system. Moreover, unlike prototype learning methods such as FedProto [44] that transmit prototypes to avoid privacy leakage (prototypes are mapped from local data based on class information), MN-FGAGN avoids this issue. Transmitting prototypes can reveal some information about the local data’s class distribution. In contrast, our method trains the discriminator locally on the client side and trains the generator on the server side, transmitting only the local discriminator’s loss value to guide the central generator’s training without transmitting specific data or model parameters. This inherently mitigates the risk of privacy leakage.

#### 4.6.2. Real-World Applications and Impact of MN-FGAGN

MN-FGAGN demonstrates broad application prospects in areas such as financial risk control, healthcare, social networks, and blockchain, especially in scenarios where data privacy is a concern and data are distributed across multiple nodes. It enables the generation of missing neighbor nodes that align with the global distribution without exposing raw data, thereby enhancing the performance of federated learning models. For instance, in financial fraud detection, it mitigates the issue of incomplete transaction networks due to privacy constraints, improving fraud identification accuracy. In the medical field, it facilitates collaborative disease prediction model training among different hospitals without sharing sensitive electronic health records. Additionally, MN-FGAGN optimizes recommendation systems for more accurate personalized recommendations and enhances the comprehensiveness of blockchain data analysis in decentralized networks. Its key advantages include improving data completeness, strengthening privacy protection, and promoting cross-institutional data collaboration, making it a significant breakthrough in federated graph learning.

#### 4.6.3. Complexity and Scalability Analysis

Through the analysis of the layer structures (See Section 5.1), the spatial complexity is determined by the number of parameters in each layer, which is calculated as the product of the input dimension and the output dimension. Therefore, the time complexity of each layer in Localgen is O(n×D×H), and the spatial complexity is O(D×H+H), where *n* denotes the number of samples, *D* represents the input dimension, and *H* indicates the number of hidden layer neurons. The time and spatial complexity of FGAGN depend on the specific structures of the generator and discriminator. In terms of model scalability, MN-FGAGN may encounter challenges when processing large-scale graph datasets. This is because Localgen requires GNN computation to be performed on client devices. If the computational power of the devices is limited, it may affect the overall training efficiency. However, MN-FGAGN supports parallel training across clients, which means that increasing the number of clients can help improve collaborative training on large-scale graph datasets.

## 5. Experiments

In this section, we discuss the experiments performed on four distinct real-world datasets to assess the efficacy of our proposed methodology across various scenarios. These datasets encompass a diverse array of application domains and incorporate different data types. To guarantee fairness and reproducibility in our experimental process, we have meticulously controlled and standardized the procedure.

### 5.1. Experimental Setup

**Datasets:** We utilized the Cora [45], Citeseer [46], and PubMed [47] datasets, which are academic paper citation networks for paper classification and graph convolutional network research in the fields of machine learning, computer science, and biomedicine. These datasets consist of papers (nodes), citation relationships (edges), and bag-of-words feature vectors. Table 2 illustrates that Cora contains 2708 papers divided into seven categories, Citeseer consists of 3327 papers divided into six categories, and PubMed comprises 19,717 papers divided into three categories. The Coauthor CS [48] dataset represents author collaboration relationships in computer science, including 18,333 authors (nodes) and 327,576 collaborations (edges). It is utilized for tasks such as social network analysis, community detection, and link prediction.

**Model training settings:** We adopted the following training settings for our model. Initially, the dataset is partitioned into a collection of subgraphs (s = 5, 10, 15) using a community detection algorithm [49] based on modularity maximization. Each subgraph is then assigned to a client. The experiments employ the Adam optimizer, with a batch size of 64 and a learning rate of 0.001. The detailed settings of the network in the experiment can be seen in Table 3, where *D* indicates the dimension of the input data.

For local generator training (localgen), we further divided the subgraph data in a 5:3:2 ratio. This split includes five unmasked subgraphs, three masked subgraphs, and two test datasets. Localgen utilizes a masked subgraph ratio of *h* = 0.15 as ground truth to predict the remaining masked data. Each training round consists of 10 epochs, and the fully trained localgen is employed to predict and repair the missing subgraph information. Subsequently, GraphSAGE samples the repaired subgraphs within the [5, 5] range and trains for two epochs.

In the federated generative adversarial network (FGAGN), the central generator produces *T*×$N_{g}^{s}$ dimensional feature data using Gaussian noise as the background. The local discriminator discriminates the data, considering local subgraphs as the ground truth, and outputs the probability that the input data are real. Throughout the training process, the discriminator is trained $D_{epoch}=5$ times after training the generator once, with the total number of generator training rounds being $G_{epoch}=200$.

**Implementation platform and experimental factor control:** All experiments were implemented using Pytorch 1.8.0+cu111 and conducted on an NVIDIA GeForce 3080 server with eight blocks of 16 GB RAM. We obtained the baseline paper’s code from GitHub and adhered to the paper’s optimal settings (e.g., original graph structure partitioning, training/testing partitioning, network dimensionality, and training process) to ensure optimal results.

### 5.2. Comparison with Alternative Methods

**Figure 6:**
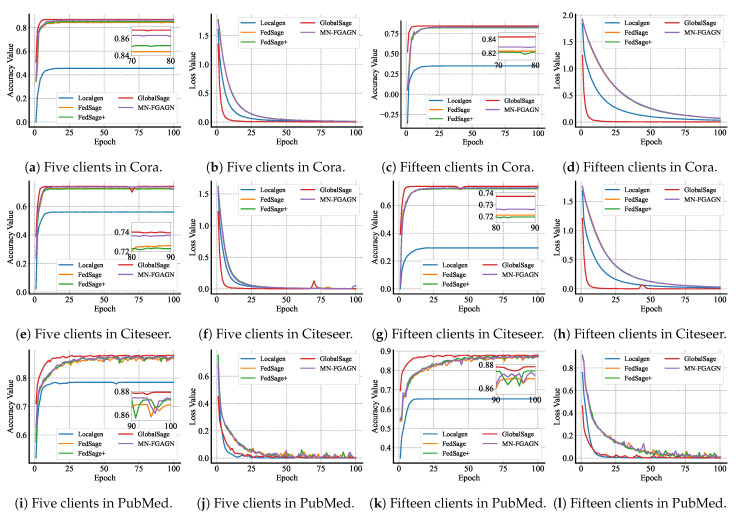
We compared the average test accuracy of MN-FGAGN with a baseline model across different client settings (10 and 15 clients) on the Cora, Citeseer, and PubMed datasets. Both models were trained for 100 epochs. The figure on the left shows the legend for the 10-client setting, while the figure on the right shows the legend for the 15-client setting.

We compare our method to the following prevalent techniques, and the specific training accuracy and loss for each method are depicted in Figure 6:

- **GlobalSage:** This method assumes that the data are centralized, and a Global GraphSAGE [43] is trained on the original global graph data, which is constructed similarly to our subgraph construction. The accuracy of this model’s test represents the training accuracy limit of the model.
- **Localgen:** Localgen first performs local repair on the subgraphs and then evaluates the Local GraphSAGE node classification accuracy using the repaired subgraph data.
- **FedSage:** This algorithm, based on FedAvg [40], trains a GraphSAGE model to learn node features, edge structures, and task labels for multiple local subgraphs. It employs GraphSAGE as a node classifier. Unlike GCN, which samples all neighboring nodes to obtain node embeddings, GraphSAGE only samples a fixed number of neighbors, significantly reducing memory consumption [50]. FedSage outperforms FedAvg in processing graph data and classifying nodes for graphs.
- **FedSage+**: As a representative method for subgraph information repair, FedSage+ enhances FedSage by training a generator that creates missing neighbors. It is trained using structurally adjacent devices on the topology to improve the generalization ability of the generator. The generator’s loss function is defined as $L=CE\left(f_{i},f_{j}\right)+CE\left(m_{i},m_{j}\right)+CE\left(l_{i},l_{j}\right)$, where i,j denote the topological adjacency of devices *i* and *j*, respectively, $f_{i}$ and $f_{j}$ represent the features of the missing nodes for devices *i* and *j*, respectively, $m_{i}$ and $m_{j}$ denote the degree of missing nodes for devices *i* and *j*, respectively, and $l_{i}$ and $l_{j}$ represent the labels of the missing information for devices *i* and *j*, respectively.

### 5.3. Performance Evaluation

**Figure 7:**
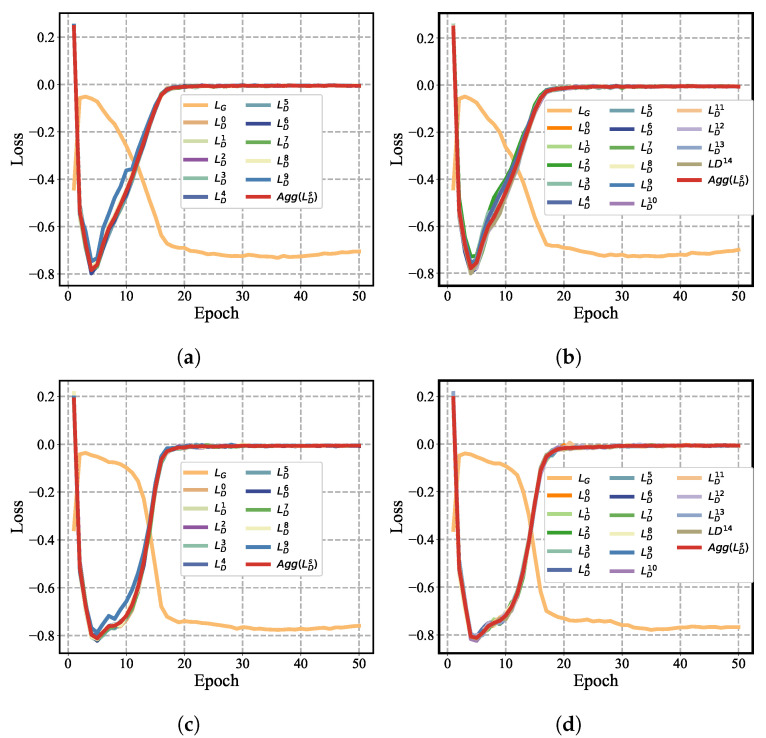
Comparison of the convergence of the generator and discriminator for different clients and different datasets. (**a**) Ten clients in the Cora dataset. (**b**) Fifteen clients in the Cora dataset. (**c**) Ten clients in the PubMed dataset. (**d**) Fifteen clients in the PubMed dataset.

**FGAGN:** In this study, we systematically evaluated the performance of the federated generative adversarial network (FGAGN) model under various scenarios, including different numbers of clients and datasets with varying levels of complexity. We primarily focused on the Cora and Citeseer datasets to establish the model’s effectiveness and robustness. The results, which include the loss functions of the FGAGN model, are illustrated in Figure 7, where Figure 7a,b represent the results for the Cora and Citeseer datasets, respectively.

As the number of clients increases, we observe that the discriminator loss function values, denoted by $L_{D}^{s}$, remain bound around the aggregated value, $Agg(L_{D}^{s})$. This indicates that the model maintains stability with increasing numbers of clients. Furthermore, as the number of training rounds increases, the generator’s loss function values, represented by $L_{G}$, and the discriminator loss function values, $L_{D}^{s}$, both converge to a stable state. This convergence suggests that the generator effectively learns the data distribution of different clients and is capable of generating regularized data with global features based on the learned content.

The generated regularized data are a mixture of multiple clients’ data, which mitigates the risk of privacy leakage. By comparing Figure 7a,b, we can infer that as the complexity of the dataset increases, the FGAGN generator’s $L_{G}$ and the discriminator loss function value $L_{D}^{s}$ maintain good convergence performance, indicating that the model is resilient to varying levels of complexity.

In conclusion, this subsection demonstrates that the FGAGN model can ensure satisfactory performance under specific conditions, such as a certain level of dataset complexity and a specified number of clients. This performance evaluation highlights the potential for the FGAGN model to be applied in federated learning scenarios, where privacy preservation and robustness against varying data distributions are crucial.

**Results and analysis:**Table 4 provides a comprehensive summary of the classification performance achieved by various methods on four real graph datasets. The test accuracy and test precision of all methods on the Cora, Citeseer, and PubMed datasets are illustrated in Figure 6, where the clients are divided into groups of 10 and 15. In this context, Localgen denotes the node classification performance on GraphSAGE after local subgraphs have been repaired by local generators.

**Figure 8:**
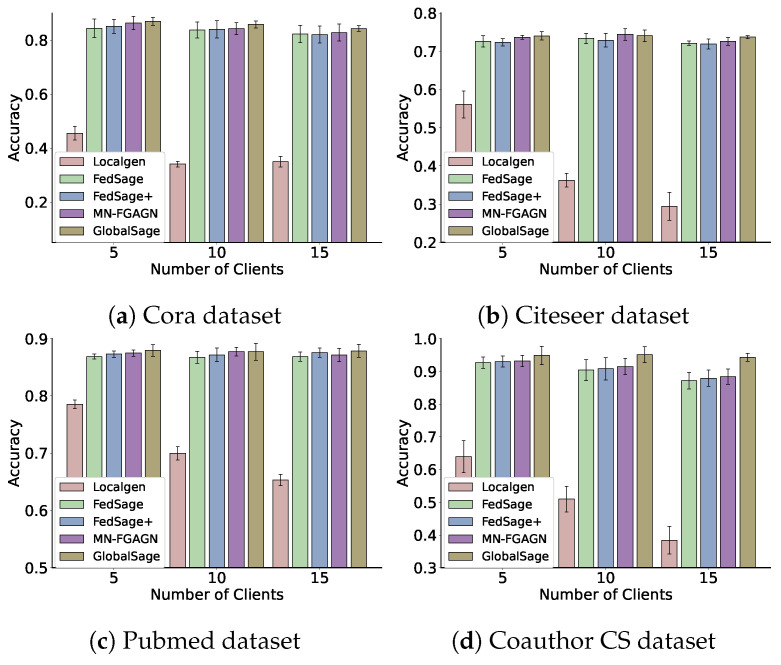
The testing accuracy distribution across all clients.

Upon analyzing the data presented in the Figure 8, it is evident that the test accuracy of Localgen is generally inferior to that of other methods. Furthermore, the accuracy of Localgen exhibits a gradual decline as the subgraph data decreases (subgraphs are divided into s = 5, 10, 15). This phenomenon can be attributed to the biased nature of the restored subgraphs and the data islanding property, which fail to provide adequate data for effective training.

FedSage, which adopts the FedAvg strategy for aggregating Localgen, demonstrates an improvement in test accuracy as the local model benefits from the local data of other clients. FedSage+ acknowledges the biased data generated by Localgen and consequently employs jointly trained Localgen-generated data to mitigate the issue of biased generated data. However, FedSage+ necessitates the broadcasting of generated data from all clients for the joint training of Localgen, which inadvertently leads to privacy leakage (since Localgen-generated data contain features of local subgraphs). Moreover, this method does not fully account for the topological connections between subgraphs, resulting in the introduction of a certain degree of noise.

Our proposed method exhibits a more than 2% increase in accuracy compared to FedSage+, which is particularly noteworthy for two reasons. Firstly, the missing subgraph data constitute only a minor portion of the total subgraph data, which implies that it should not exert a disproportionate influence. However, this improvement is still significant within the context of federated graph learning research. Secondly, our method employs a novel distributed generation network to directly generate data containing global features. These data are then utilized as regularized data to eliminate the bias inherent in Localgen-generated data. This approach obviates the need for transmitting Localgen generation nodes, thereby offering superior privacy-preserving performance. Consequently, it represents a promising new research direction for addressing the cross-subgraph neighborhood missing problem in federated graph learning.

**Visual explanation of node classification performance:** In order to thoroughly assess the efficacy of MN-FGAGN in the context of node classification, we present a visual examination of the embedding spaces generated by two essential components, namely, Localgen and FGAGN, during the classification process. This approach allows for a more intuitive and comprehensive understanding of their individual and combined performance.

By meticulously inspecting the visual representations of the embeddings generated by both Localgen and FGAGN, we can deduce that these two components are capable of effectively classifying nodes after undergoing the training process, subsequently resulting in favorable outcomes. This observation not only provides evidence for the validity of the proposed model but also helps to elucidate the inner workings of its components.

**Figure 9:**
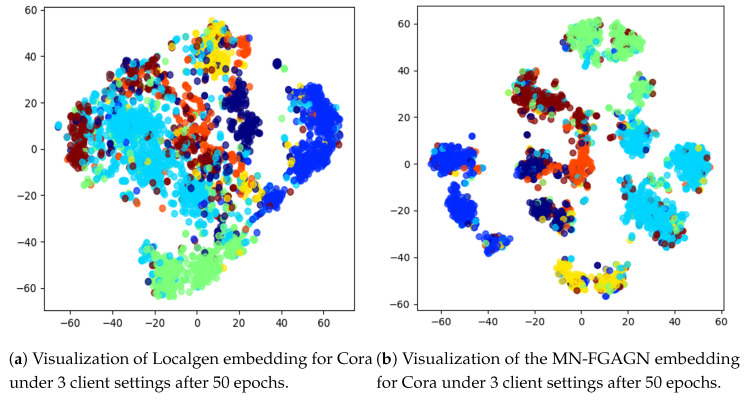
(**a**) The node classification results of Localgen, and (**b**) the node classification results of FGAGN, where the seven colors correspond to the seven categories of the Cora dataset. By comparison, we can find that the node classification effect of FGAGN is better than that of Localgen, which is reflected by the fact that FGAGN has more cohesive clustering in the figure.

However, upon further examination, it becomes apparent that FGAGN exhibits a higher degree of efficiency in terms of classification performance when compared to Localgen. This superior performance is vividly illustrated in Figure 9b, which displays a more pronounced and cohesive clustering of nodes in the embedding space as opposed to the relatively less distinct clustering observed in Figure 9a. This finding highlights the pivotal role that FGAGN plays in the overall performance of MN-FGAGN, particularly in node classification tasks, and substantiates its relevance as a major contributor to the success of the proposed model.

### 5.4. Ablation Experiment

In this comprehensive ablation study, we rigorously investigate the individual and combined influences of the local generation module (Localgen) and the federated graph neural generation adversarial neural network module (FGAGN) on the overall performance of the proposed MN-FGAGN model. We conduct experiments using four prominent benchmark datasets: Cora, Citeseer, PubMed, and Coauthor CS. The outcomes of these experiments are presented in Table 5, which details the performance metrics of different MN-FGAGN variants on each dataset. A comparative analysis of variants 1 and 2 enables us to discern the impact of integrating both modules on the model’s performance.

Our empirical findings unveil that employing either the standalone Localgen module or the FGAGN module independently does not suffice in effectively learning the data distribution for each client. A closer examination of the results, specifically comparing variant 1 with the full MN-FGAGN model and variant 2 with the full MN-FGAGN model, allows us to deduce that the FGAGN module contributes more significantly to the overall performance of the model than the Localgen module. This observation can be primarily attributed to the fact that the Localgen module is designed to focus on learning local subgraph features, thereby serving predominantly as an indirect, learnable target for the FGAGN module. In stark contrast, the FGAGN module is tailored to capture global graph features, which empowers it to learn more accurate and robust feature representations.

Ultimately, our analysis confirms that the MN-FGAGN model, which incorporates both the Localgen and FGAGN modules, exhibits the most superior performance across all datasets. This finding underscores the notion that the modules exhibit a synergistic relationship, effectively complementing and enhancing each other’s contributions. The observed synergy between the Localgen and FGAGN modules accentuates their indispensability in the construction of the MN-FGAGN model and further reinforces the efficacy of our proposed approach.

### 5.5. Parameters Sensitivity Analysis

**Figure 10:**
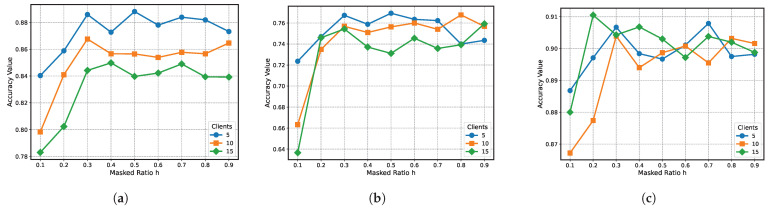
Effect of masked ratio on classification accuracy across different datasets and client numbers. (**a**) Masked ratio *h* of MN-FGAGN in Cora. (**b**) Masked ratio *h* of MN-FGAGN in Citeseer. (**c**) Masked ratio *h* of MN-FGAGN in PubMed.

To analyze the effect of the hyperparameter *h* (representing the proportion of masked local subgraphs) on the performance of the MN-FGAGN model, we conducted a series of systematic experiments to explore how different values of *h* (ranging from 0.1 to 1) affect the model’s accuracy. As shown in Figure 10, we observed that changes in the value of *h* significantly influence model performance. Notably, when *h* exceeds 0.3, the model’s accuracy tends to stabilize.

Specifically, our analysis involved the following steps:

1. **Systematic grid search:** We tested various values of *h* (0.1, 0.2, 0.3, …, 1) on multiple datasets and calculated the model’s classification accuracy to determine the optimal range of *h*.
2. **Stability analysis:** To ensure the reliability of the experimental results, we repeated the experiments under different random seeds and data splits to assess whether the effect of *h* on model performance remained consistent.
3. **Cross-dataset validation:** We conducted experiments across multiple datasets to verify the general applicability of the empirical value h=0.3.

Figure 10 graphically demonstrates the relationship between the accuracy of the MN-FGAGN model and the *h* value. It can be observed that the model’s accuracy exhibits a positive correlation with the *h* value, as it progressively increases with the rising proportion of masked local subgraphs. However, this upward trend plateaus once the *h* value reaches approximately 0.3, beyond which the model’s score experiences slight fluctuations around a specific level without a substantial improvement in accuracy. This empirical analysis indicates that an overly small *h* value may not be conducive to generating more accurate augmentation data for local subgraphs, which in turn can adversely affect the global regularized data generator’s capacity to learn the local node distribution effectively. Consequently, it is crucial to fine-tune the hyperparameter *h* to identify the optimal value that yields the highest node classification scores for the Cora and Citeseer datasets. Our findings suggest that an *h* value of approximately 0.3 strikes a balance between adequate masking of local subgraphs and preserving the model’s ability to learn from the global regularized data generator, thus achieving the best overall performance.

## 6. Conclusions

In the field of cross-domain subgraph federated learning, current algorithms often generate missing nodes through local subgraphs, leading to the presence of node biases. To overcome this issue, we present a novel federated graph learning framework, which addresses locally biased nodes by incorporating a federated generative neural network. This network guides a server-side generator to produce regularized data that effectively correct locally generated node biases. Our approach offers two key benefits: (i) it eliminates the need for clients to directly share sensitive information regarding missing nodes, thereby preserving privacy and only providing the necessary information to adjust local data; (ii) it improves the quality of the global model without transmitting local data, generating missing neighbor information with global characteristics and improving the quality of local subgraph data. Our experiments on four datasets demonstrate that MN-FGAGN outperforms existing state-of-the-art baselines under comparable conditions, thus affirming the efficacy of our method.

## 7. Future Work

In future work, we plan to further extend the applicability of MN-FGAGN to address more complex federated graph learning scenarios. Currently, MN-FGAGN primarily focuses on correcting the bias in local subgraph data by generating pseudo-nodes that better align with the global distribution, thereby enhancing the overall model performance. However, real-world federated graph learning often presents additional challenges, particularly in two key areas: First, addressing the heterogeneity of subgraphs across different clients. Specifically, the structural and feature distribution differences among client subgraphs require an optimized global generation strategy. This would enable the generated pseudo-nodes to adapt to heterogeneous subgraphs, thereby mitigating the negative impact of distribution imbalance on model performance. Second, extending MN-FGAGN to dynamic federated spatiotemporal graph learning. This involves adapting to time-varying graph structures in applications such as recommendation systems and traffic flow prediction. By leveraging temporal information, the model can refine the generation strategy for missing neighbors, thereby improving its ability to learn and adapt to evolving patterns over time.

## Figures and Tables

**Table 1:** A summary of main mathematical symbols.

Symbol	Definition	Symbol	Definition
$N_{c}$	Number of clients	$N_{g}^{n}$	Number of missing nodes
*S*	Central server of MN-FGAG		generated by the local generator
$G_{global}$	Server-side global generator	$N_{mask}$	Degree of masked nodes
$D_{n}$	Discriminator of client *n*	$V_{mask}$	Masked nodes
G	Global undirected graph	$N_{pred}$	Degree of predicted missing nodes
*V*	Set of nodes		
*F*	Node feature	$V_{pred}$	Predicted missing nodes
*E*	Set of edges	$N_{g}^{all}$	Total number of missing nodes
β	Set of selected regularized data		across all clients
α	Set of locally generated	*h*	Masked ratio of local graphs
	biased nodes	$F_{new}$	New feature after regularization
d(·)	Feature distance calculation	$\omega_{n}$	Weight factor for combining local
	function		and regularized data

**Table 2:** Graph of specific statistics of datasets.

Datasets	Cora	Citeseer	Pubmed	Coauthor CS
Nodes	2708	3312	19,717	34,493
Edges	5429	4715	44,338	247,962
Classes	7	6	3	5
Dimension	1433	3703	500	8415

**Table 3:** Network architecture of the Localgen, global generator, and discriminator.

Layer	Details (Localgen)	Layer	Details (Generator)	Layer	Details (Discriminator)
1	G-conv (D, 128) + RELU	1	Random-noise (D)	1	Linear (D, 128) + RELU
2	G-conv (128, 64) + RELU	2	Linear (D, 128) + RELU	2	Linear (128, 256) + RELU
3	FC (64, 1) + Sigmoid	3	Linear (128, D) + tanh	3	Linear (256, 1) + Sigmoid
4	Random-noise (64)				
5	FC (64, 256) + RELU				
6	FC (256, D) + tanh				

**Table 4:** The experimental results of node classification on four different datasets with $N_{c}$ = 5, 10, and 15 clients provide the corresponding accuracy of different methods.

Datasets	Cora			Citeseer		
Methods	$N_{c}$ = 5	$N_{c}$ = 10	$N_{c}$ = 15	$N_{c}$=5	$N_{c}$ = 10	$N_{c}$ = 15
Global Model	87.01	85.87	84.32	74.04	75.75	73.71
	(±0.0143)	(±0.0129)	(±0.0104)	(±0.0109)	(±0.0085)	(±0.004)
LocalSage	45.49	34.04	34.95	56.08	36.26	29.45
	(±0.0255)	(±0.0101)	(±0.0200)	(±0.0351)	(±0.0176)	(±0.0367)
FedSage	84.44	83.81	82.28	72.35	72.90	71.95
	(±0.0349)	(±0.0296)	(±0.0325)	(±0.0093)	(±0.0181)	(±0.0131)
FedSage+	85.15	84.05	82.11	72.61	73.35	72.10
	(±0.0256)	(±0.0324)	(±0.0312)	(±0.0151)	(±0.0138)	(±0.0057)
MN-GAGN	**86.37**	**84.30**	**82.86**	**73.67**	**74.40**	**72.61**
	**(±0.0244)**	**(±0.0219)**	**(±0.0316)**	**(±0.0056)**	**(±0.0156)**	**(±0.0107)**
Datasets	PubMed			Coauthor CS		
Methods	$N_{c}$ = 5	$N_{c}$ = 10	$N_{c}$ = 15	$N_{c}$ = 5	$N_{c}$ = 10	$N_{c}$ = 15
Global Model	87.93	87.70	87.84	94.84	95.10	94.22
	(±0.0103)	(±0.0149)	(±0.0114)	(±0.0279)	(±0.0242)	(±0.0122)
LocalSage	78.52	69.95	65.31	63.94	50.95	38.43
	(±0.0074)	(±0.0117)	(±0.0101)	(±0.0487)	(±0.0388)	(±0.0419)
FedSage	86.87	86.74	86.85	92.69	90.36	87.15
	(±0.0046)	(±0.0106)	(±0.0081)	(±0.0173)	(±0.032)	(±0.0258)
FedSage+	87.28	87.15	87.13	92.96	90.76	87.90
	(±0.0055)	(±0.0119)	(±0.0116)	(±0.0167)	(±0.0337)	(±0.0247)
MN-GAGN	**87.46**	**87.72**	**87.52**	**93.12**	**91.46**	**88.37**
	**(±0.0054)**	**(±0.0076)**	**(±0.0081)**	**(±0.0174)**	**(±0.0252)**	**(±0.0230)**

**Table 5:** The ablation experiment results of node classification on 4 different datasets of N = 5, 10, and 15 clients show that the accuracy of only the Localgen model or split GAN model is lower than that of MN-FGAGN.

Datasets	Localgen	FGAGN	Cora			Citeseer		
Methods			$N_{c}$ = 5	$N_{c}$ = 10	$N_{c}$ = 15	$N_{c}$ = 5	$N_{c}$ = 10	$N_{c}$ = 15
Variant 1	✔	-	68.52	54.72	51.43	66.17	56.72	52.27
Variant 2	-	✔	83.49	84.04	79.42	76.62	76.65	73.88
MN-FGAGN	✔	✔	**83.67**	**82.45**	**79.38**	**76.09**	**76.20**	**73.84**
Global Model	✔	✔	85.42	85.92	83.30	76.02	76.35	74.14
Datasets	Localgen	FGAGN	PubMed			Coauthor CS		
Methods			$N_{c}$ = 5	$N_{c}$ = 10	$N_{c}$ = 15	$N_{c}$ = 5	$N_{c}$ = 10	$N_{c}$ = 15
Variant 1	✔	-	83.86	77.47	74.24	87.18	86.82	86.17
Variant 2	-	✔	88.15	87.20	86.76	91.67	88.34	85.33
MN-FGAGN	✔	✔	**88.00**	**88.40**	**88.72**	**91.89**	**89.67**	**86.74**
Global Model	✔	✔	89.01	89.41	89.14	92.87	93.39	93.36

## Data Availability

Data are contained within the article.
